# Olfactory Cues and Patient Safety Behavior in Virtual Reality Medical Simulation: Controlled Trial

**DOI:** 10.2196/93148

**Published:** 2026-08-19

**Authors:** Anna Junga, Pascal Kockwelp, Jennifer Dabel, Sönke Helmut Scherzer, Benjamin Risse, Markus Holling, Bernhard Marschall, Hendrik Friederichs

**Affiliations:** 1 Institute of Education and Student Affairs, University of Münster, Albert-Schweitzer-Campus 1A, Building A6, Münster, North Rhine-Westphalia, 48149, Germany, 49 25183-41133, 49 25183-58933; 2 Institute for Geoinformatics & Faculty of Mathematics and Computer Science, University of Münster, Münster, North Rhine-Westphalia, Germany; 3 Department of Neurosurgery, University Hospital Münster, Münster, North Rhine-Westphalia, Germany; 4 Research Group Medical Education, Medical School OWL, Bielefeld University, Bielefeld, North Rhine-Westphalia, Germany

**Keywords:** virtual reality, undergraduate medical education, hand hygiene, olfactory, patient safety, simulation, immersion, brain death

## Abstract

**Background:**

Simulation-based medical education is essential for improving patient safety. In virtual reality (VR)–based simulation, immersion is primarily generated through visual and auditory cues, while other sensory modalities are typically absent. This sensory limitation may reduce the emergence of authentic safety-relevant behaviors. Olfaction plays an important role in clinical reasoning, risk perception, and self-protective behavior and is closely linked to memory and emotion. Although olfactory cues have been shown to influence hand hygiene behavior in real or simulated environments, their targeted integration into fully immersive VR-based medical simulation has not been systematically examined.

**Objective:**

This study aimed to investigate whether adding a real olfactory cue (disinfectant scent) to a fully virtual clinical simulation increases patient safety–relevant behavior, specifically hand hygiene compliance (hand disinfection and glove usage).

**Methods:**

In a controlled study at the University of Münster (during the winter term 2025-2026), 95 medical students participated in a VR-based clinical simulation. Study rooms were preassigned to either an olfactory intervention or a control condition, and participants selected their room without knowledge of the assigned condition (quasi-random allocation by room). Hand hygiene and glove use were automatically tracked as outcomes. Odds ratios (ORs) were calculated to assess the effect of the intervention on these behaviors.

**Results:**

The olfactory intervention nearly tripled the odds of hand disinfection (8/43, 18.6% vs 20/52, 38.5%; an absolute risk increase of 19.9 percentage points; OR=2.73, 95% CI 1.09‐7.40; *P*=.04; number needed to treat=5), while no significant difference was observed for glove use (OR=1.44, 95% CI 0.62‐3.45; *P*=.40).

**Conclusions:**

The integration of a real olfactory cue into a fully immersive VR medical simulation significantly increased hand disinfection behavior, particularly after patient contact, but did not affect glove use. These findings suggest that olfactory augmentation can selectively reinforce safety-relevant behaviors in digital training environments. Incorporating real-world sensory cues into VR may represent a simple yet effective design strategy to enhance behavioral authenticity and patient safety outcomes in simulation-based medical education.

## Introduction

Simulation in medical education has become an essential strategy for improving health care quality and, most importantly, patient safety. Modern simulation modalities—ranging from manikins and standardized patients to fully immersive virtual reality (VR) environments—enable learners to practice technical skills, develop situational awareness, and recognize safety-relevant cues in a controlled, risk-free setting. Effective simulation is thus not only about skill acquisition but also about fostering behaviors that prevent harm to patients.

Across all simulation modalities, immersion—the subjective perception of a simulated environment as “real”—is critical for learner engagement and the transfer of knowledge to clinical practice. It is useful to distinguish immersion, the objective sensory and technical fidelity of the system, from presence, the subjective sense of being there, and from embodiment, the experience of the virtual body as one’s own. These constructs are conceptually distinct but jointly shape how learners act in virtual environments. Immersion arises from the integration of visual, auditory, tactile, and contextual cues, with the surrounding environment often contributing substantially in traditional simulation centers. Purpose-built clinical simulation environments aim to replicate real-world contexts as faithfully as possible, integrating multiple sensory inputs to strengthen both realism and learner engagement [1,2]. Hybrid solutions, such as SimuScape [3], have further demonstrated that combining real interaction partners with projected environments can enhance learning while remaining economically feasible.

VR-based simulation, in contrast, creates immersion almost entirely through digitally generated environments, enabling learners to actively explore and interact with complex clinical situations rather than passively observe them. This interactivity is enabled by head-mounted displays (HMDs) and hand-tracking technologies. However, the sensory experience in VR is largely restricted to virtual visual and auditory cues, while tactile feedback remains limited and other sensory modalities are typically absent [4-6]. As a result, clinically relevant safety behaviors may not reliably emerge, even when scenarios are cognitively well-designed. Previous work by Junga et al [7,8] indicates that, in VR-based simulations, hand hygiene–related behavioral targets are not consistently achieved, presumably because the level of situational realism is insufficient to trigger safety-relevant actions.

This raises a central design challenge for VR-based medical education: How can digital simulations be augmented to elicit authentic, real-world patient safety behavior? In this context, olfactory cues are of particular interest. Unlike visual or auditory stimuli in VR, there is no widely used technology to render smell, so it must originate from the physical world. Olfaction, therefore, represents the only manipulatable sensory input in VR-based simulations that is entirely real. Introducing real smells, such as disinfectants, into a VR scenario may help learners cross the virtual threshold, experiencing the situation in a way that more closely resembles clinical practice and thereby increasing the likelihood of authentic patient safety behavior.

Evidence from cognitive psychology suggests that learning and recall are enhanced when the sensory context of learning closely resembles that of application, a phenomenon known as context-dependent memory [9]. In clinical practice, olfactory cues play a multifaceted role in patient safety and clinical decision-making. They may provide diagnostic information (eg, ketone odor in diabetic ketoacidosis or alcohol odor in unconscious patients), signal environmental or personal risk (eg, gas leaks or intoxication-associated aggression), and support rapid situational assessment. Olfaction is also closely linked to memory formation and emotional processing, making it a powerful but often implicit driver of behavior.

Despite this, olfactory input has rarely been integrated into VR-based medical education. For non-VR simulation, Birnbach et al [10] conducted a landmark randomized study in 2013 investigating the effect of a fresh citrus scent on hand hygiene compliance. In the control group (n=86), the compliance rate was 51%, whereas the scent group (n=79) achieved a significantly higher rate of 80% (*P*<.001). A subsequent study by King et al [11] confirmed these findings in a surgical intensive care unit. Exposure to a citrus scent resulted in improved hand hygiene compliance (46.9% vs 15.0%; *P*<.001), corresponding to an odds ratio (OR) of approximately 5.0. In contrast, Schmidtke et al [12] reported differing results. In a large crossover study conducted at hospital ward entrances, none of the priming interventions led to a consistent increase in hand hygiene behavior.

Notably, all previous studies investigating olfactory effects on hand hygiene were conducted in real-world or simulated environments. To date, the targeted integration of real olfactory cues into fully immersive VR-based medical simulation has not been systematically examined. Building on our previous findings showing that immersion levels and the visual representation of virtual hands can measurably influence hygiene behavior [7,8], the present study investigates whether adding real olfactory cues to an otherwise fully virtual environment can enhance hand hygiene practices, including prepatient and postpatient contact disinfection.

By conceptualizing olfactory augmentation as a design element in digital simulation rather than a contextual add-on, this study addresses a critical gap in VR-based medical education. Based on the heterogeneous evidence with positive findings from controlled simulation environments and negative findings from real-world settings, we hypothesize that exposure to real olfactory cues (disinfectant scent) during a VR simulation with defined patient contact increases hand hygiene behavior compared with a VR simulation without olfactory augmentation. As a second primary outcome, we examine whether the olfactory intervention also influences glove use.

## Methods

### Setting and Participants

The study was carried out as part of the “Brain Death/Death by Neurologic Criteria” (BD/DNC) examination course at the University of Münster during the winter term 2025-2026. The course is scheduled for the seventh semester of the medical curriculum. A VR-based digital simulation depicting a clinical scenario for BD/DNC examination served as the research environment [13-15]. The VR environment replicates a realistic intensive care unit in which participants can interact with a virtual patient and perform clinical examinations. The course is part of a block week focusing on organ donation and has been delivered in a VR-based format since 2021. Further information about the course structure and procedures can be found in Junga et al [13,16]. Every student received a technical and medical preparation video podcast to overcome knowledge and computer literacy inequalities.

Although participation in the course was mandatory, participation in the study was voluntary. Students who declined to participate did not experience any disadvantages. Students enrolled in the course were recruited and assigned to 1 of 2 conditions through quasi-random allocation by room. Rooms were preassigned to either a control condition (“NoSmell”), in which participants performed the standard brain death examination, or an intervention condition (“Smell”), in which the same simulation was augmented with an olfactory stimulus. Participants selected their room without knowledge of the assigned condition or about the assignment at all. Inclusion criteria included enrollment in medical school, course attendance, and willingness to participate in the study.

### Ethical Considerations

The study was conducted in accordance with the Declaration of Helsinki and was approved by the responsible ethics committee (Ethik-Kommission Westfalen-Lippe; application 2022‐736-b-S; approved on November 9, 2022). Participation in the study was voluntary and independent of course participation, completion, and assessment, and declining to participate entailed no disadvantage. Written informed consent was obtained from all participants prior to allocation through a digital survey form. All study data were collected and analyzed in pseudonymized form using 6-character study codes, and no directly identifying information was retained at the analysis stage. No financial or nonfinancial compensation was provided for study participation.

### Sample Size Calculation

The required sample size was determined a priori using G*Power (version 3.1; Heinrich Heine University Düsseldorf) [17]. For the primary dichotomous outcomes (hand disinfection and glove use, each coded as true or false), a medium effect size (*w*=0.30) [18] was assumed, and a *χ*^2^_1_ test for a 2×2 contingency table was specified. With an α level of .05 and a statistical power of 80%, the required total sample size was 88. To account for potential dropouts, a minimum of 90 participants was targeted for recruitment.

### Outcome Objectives and Necessary Adjustments

In accordance with the recommendations of the Commission for Hospital Hygiene and Infection Prevention (KRINKO) at the Robert Koch Institute, hand disinfection is mandatory before patient contact or contact with the patient’s environment (eg, bed, side table, and catheter bag), before aseptic procedures, and after contact with potentially contaminated material or the patient and their surroundings. The use of medical gloves is recommended for “protection of the wearer against contamination with blood, secretions, and excretions, including pathogens, and indirectly to interrupt chains of infection” [19].

**Figure 1.**
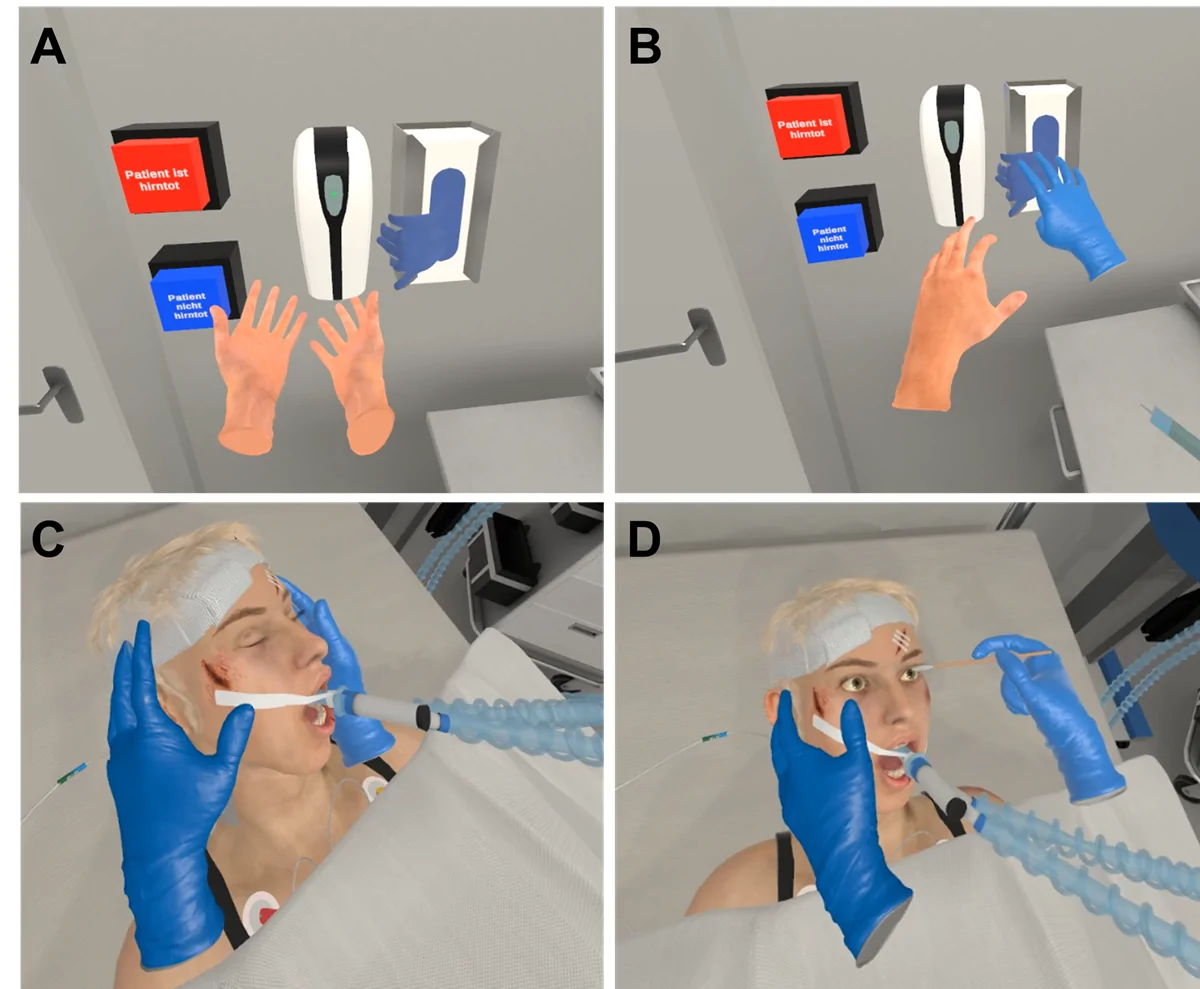
In-game screenshots of the implemented hygiene stations and clinical application. (A) Activation of the disinfectant dispenser. (B) Interaction with the glove box to equip protective gear. (C, D) Subsequent patient examination (head stabilization and corneal reflex test) performed with gloved hands.

For the purposes of this study, the functionalities for using gloves and a hand disinfectant dispenser were specifically implemented and enabled within the VR environment (Figure 1A, B). To use the dispenser, at least one hand had to be placed underneath it, and a hissing sound was generated to confirm the interaction. To use the gloves, the participant’s hands had to touch the glove box, after which the gloves were attached automatically.

The software was further modified so that, in addition to interactions associated with a potential risk of exposure to bodily fluids (eg, gag reflex, tracheal reflex, and caloric-induced nystagmus), the examination procedure deliberately involved contact with a simulated extensive skin abrasion in the facial area (Figure 1C, D). This design rendered glove use mandatory for hygiene, in addition to hand disinfection, which is strictly required in intensive care settings.

As demonstrated in Junga et al [8], the representation of the hands significantly affects glove usage. Hence, the application was modified to provide the avatar with a bare hand model, as opposed to the previous one, which was equipped with gloves by default.

The objectives stated in Table 1 were identified in accordance with the recommendations for hand hygiene issued by the Robert Koch Institute [19]. Primary and explorative outcomes were analyzed separately. All actions were automatically tracked and documented by the software. Before and after the course, participants completed a survey on demographics and prior VR experience. No item assessed scent perception or other manipulation-check questions.

All end points were operationalized as binary variables. Hand disinfection was considered to have been performed if the participant activated the virtual disinfectant dispenser with at least one hand. Glove use was recorded if the participant interacted with the glove box using at least one hand, thereby triggering the glove application within the virtual environment.

**Table 1.** Primary and explorative objectives used in this study.

Task-implicit objectives (hand hygiene)	Measure
Primary outcome	
Hand disinfection was applied overall (at least one hand)	True or false
Gloves were put on overall (at least one hand)	True or false
Explorative outcome	
Gloves were put on before first patient contact (at least one hand)	True or false
Hand disinfection was applied before first direct patient contact (at least one hand)	True or false
Hand disinfection was applied after last patient contact (at least one hand)	True or false

### Olfactory Intervention Procedure

**Figure 2.**
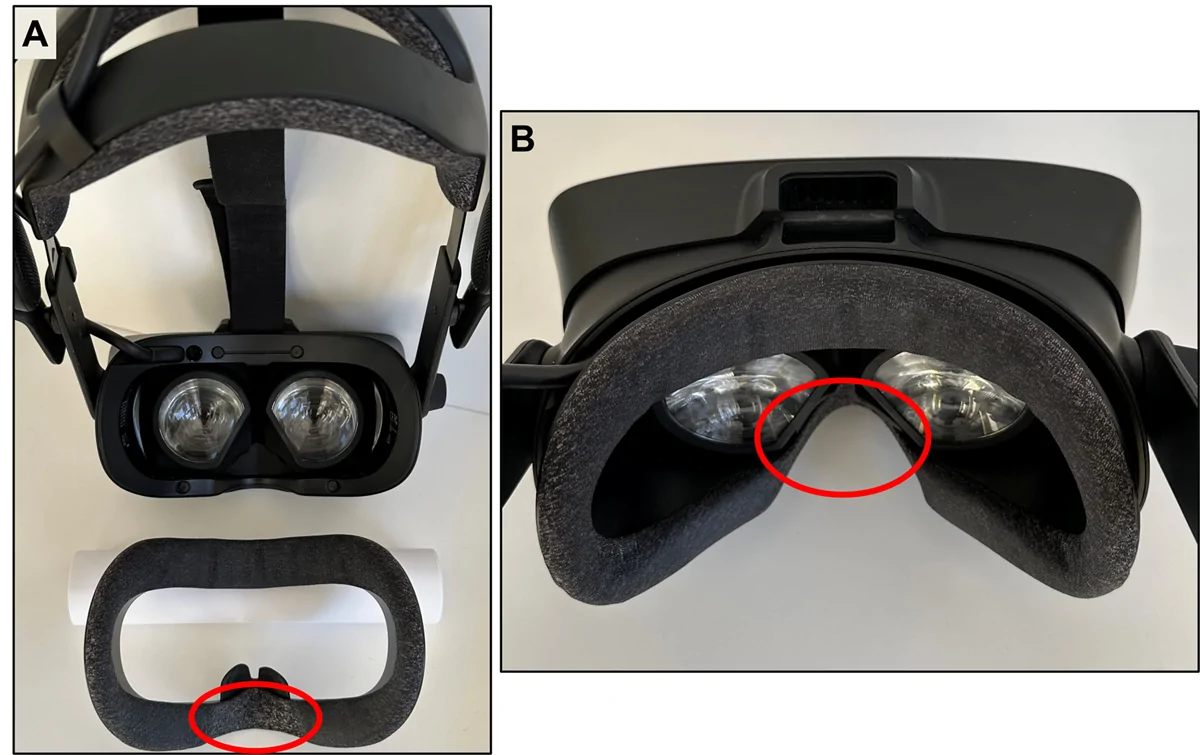
Inside views of the Valve Index virtual reality headset (view from above) showing the area of applied disinfectant on the facial interface (red). (A) Headset with facial interface removed for better visibility. (B) Headset with facial interface attached.

In 3 of the 6 rooms, an olfactory stimulus was applied by placing 5 drops (approximately 0.25 mL) of an alcohol-based hand disinfectant (desmanol care, Schülke & Mayr GmbH) onto the facial interface of the VR headset, specifically at the upper edge of the nasal cutout (Figure 2). This application was repeated immediately before each new rotation (<3 min) to avoid differences in smell intensity.

Due to the use of additional hygienic face masks, there was no direct skin contact with the students’ faces, but the fluid was placed less than 5 mm from the bridge of the nose without any visible manipulation of the HMD. Participants were blinded to both the presence and the purpose of the olfactory intervention. The use of hand disinfectants and gloves in the virtual environment was automatically tracked by the system, and the data were exported after course completion.

### Materials

The VR scenario was delivered using the *BD/DNC Examination* software (version December 2025; University of Münster) [13-15] on a Valve Index HMD with Knuckles controllers (Valve Corporation). Data were collected using LimeSurvey and analyzed with R (version 4.5.2; R Foundation for Statistical Computing) using the tidyverse [20] and broom [21] packages.

### Statistical Methods

#### Primary Analysis

The effect of the olfactory intervention on the 2 primary end points (hand disinfection and glove use) was evaluated using logistic regression on the intention-to-treat sample of all 95 allocated participants, with intervention condition entered as the sole predictor. Parameter estimation was performed using the maximum likelihood method. Results are reported as ORs with 95% CIs. In addition, standardized regression coefficients are reported, obtained by fitting the model to a standardized version of the dataset. CIs were obtained by the profile-likelihood method. Model fit was quantified using Tjur *R*^2^, a pseudo-coefficient of determination appropriate for logistic regression.

#### Sensitivity Analyses

Results were verified using Fisher exact and Pearson chi-square tests. Effect size was calculated as follows: Cohen *w* = √(*χ*²/N).

#### Exploratory End Points

Given low expected cell counts (<5) for some end points, Fisher exact test was used throughout to calculate *P* values, whereas the corresponding ORs and 95% CIs were obtained from logistic regression (with a Haldane-Anscombe correction for an empty cell in initial glove use). Exploratory end points were reported with unadjusted *P* values and should therefore be interpreted with appropriate caution.

The study was reported in accordance with the CONSORT-EHEALTH (Consolidated Standards of Reporting Trials of Electronic and Mobile Health Applications and Online Telehealth) checklist, version 1.6.1. The completed checklist is provided in Checklist 1.

## Results

### Inclusion Criteria

**Figure 3.**
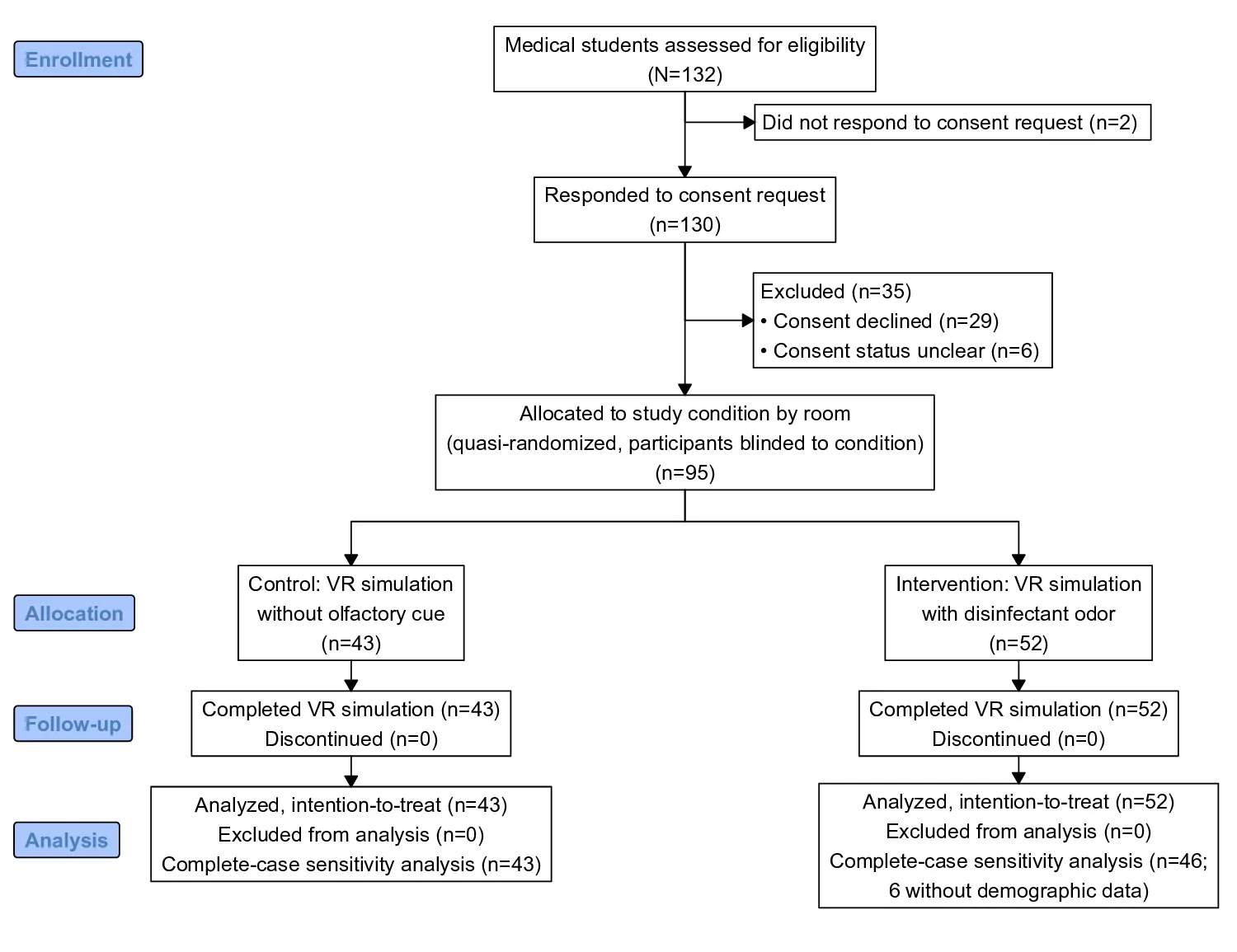
Participant flow through the study. Allocation to the intervention (olfactory cue) or control condition was performed at room level (quasi-random allocation); participants were blinded to their assigned condition. All allocated participants completed the simulation and were included in the intention-to-treat analysis. VR: virtual reality.

In the winter term 2025-2026, 132 individuals were assessed for eligibility, of which 130 responded to the consent request. Of these, 35 were excluded: 29 refused consent, and the consent status of 6 remained unclear. The remaining 95 participants were allocated to the control group (n=43) or the intervention group (n=52). All 95 allocated participants completed the VR simulation and were analyzed according to their assigned condition (intention-to-treat analysis). Six participants in the intervention group did not provide demographic data and were therefore excluded from the complete-case sensitivity analyses only (complete-case: n=89, comprising 43 control and 46 intervention participants). The minor imbalance in group sizes reflects the quasi-random allocation by room, as final numbers were determined only after all sessions had been completed. The flow of participants through the study is shown in Figure 3.

### Participant Characteristics

As shown in Table 2, participants had a mean age of 23.6 (SD 2.6; range 21‐35) years. Age did not differ significantly between conditions (24.0 vs 23.2 y; *t*_67.8_=1.51; *P*=.14). Female students (59/89, 66.3%) were overrepresented in the sample, consistent with the sex composition of medical students in Germany [22]. However, the sex distribution did not differ significantly between groups (32/43, 74.4% vs 27/46, 58.7% female participants; *χ*²_1_=2.46; *P*=.12).

In the subsequent survey about prior HMD experience, only 3 participants reported owning a personal VR headset, all of whom were male. However, the majority of students (52/89, 58.4%; regularly+once or rarely) had previously used a VR headset at least once (for further information, see Table 3).

**Table 2.** Baseline characteristics (N=89).

Characteristic	Overall (N=89)	Control (n=43)	Intervention (n=46)	*P* value
Age (y)				.14^a^
Mean (SD)	23.6 (2.6)	24.0 (3.1)	23.2 (1.9)	
Median (IQR)	23 (22-24)	23 (22–25)	23 (22–23)	
Range	21‐35	21‐35	21‐29	
Sex, n (%)				.12^b^
Female	59 (66.3)	32 (74.4)	27 (58.7)	
Male	30 (33.7)	11 (25.6)	19 (41.3)	

a Independent-samples *t* test (2-tailed, Welch correction for unequal variances).

b Pearson chi-square test (2-tailed, *df*=1).

**Table 3.** Characteristics of participants, divided into total (N=89), intervention (“Smell”, n=46), and control group (“NoSmell”, n=43).

Characteristic	Total (N=89), n (%)	Smell (n=46), n (%)	NoSmell (n=43), n (%)
Owns HMD^a^	3 (3.37)	1 (2.17)	2 (4.65)
HMD experience			
Never used	37 (41.57)	19 (41.30)	18 (41.86)
Once or rarely	50 (56.18)	27 (58.70)	23 (53.49)
Regularly	2 (2.24)	0 (0)	2 (4.65)

a HMD: head-mounted display.

### Primary Outcome

#### Hand Disinfection

**Figure 4.**
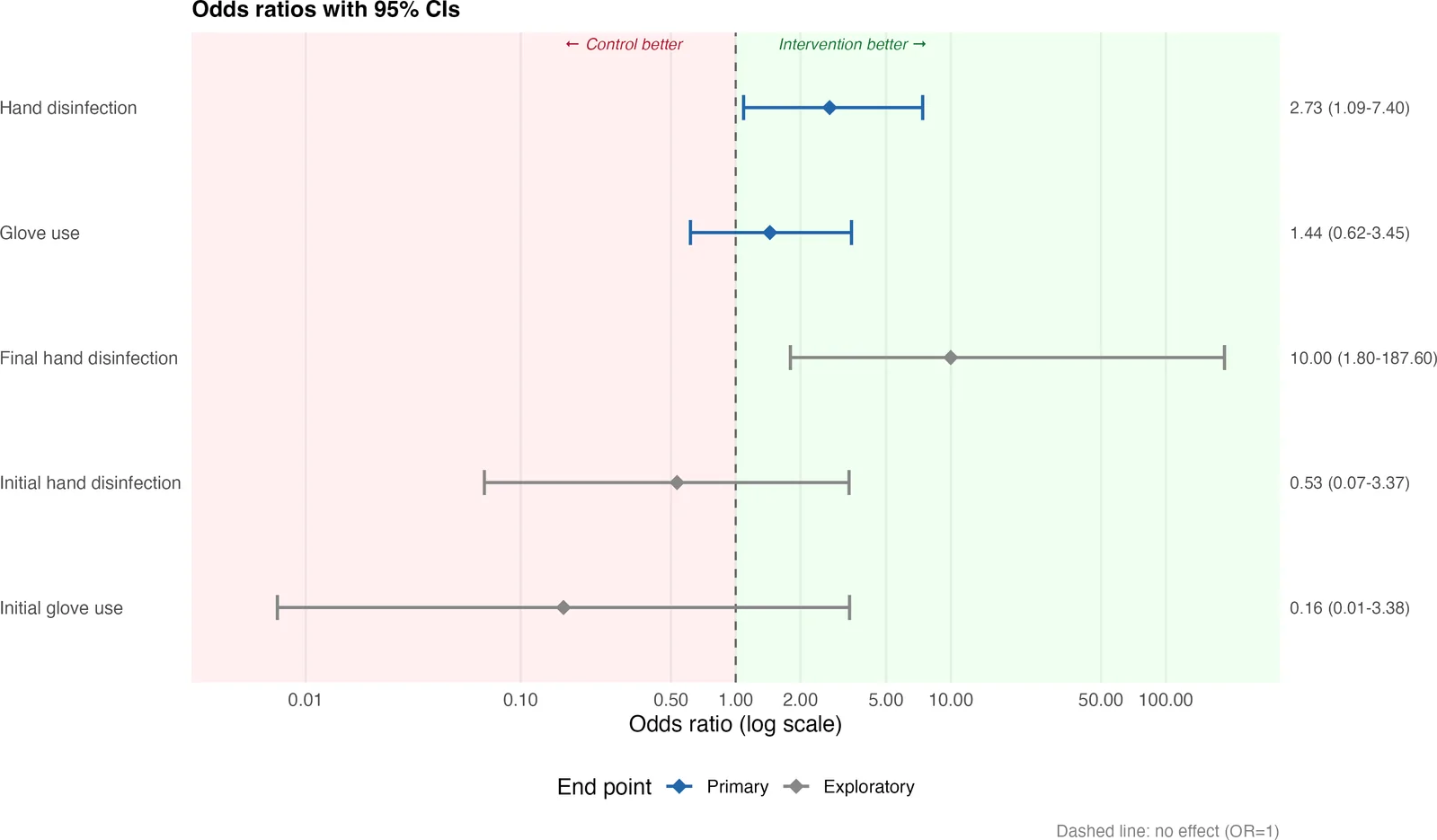
Forest plot (effect of olfactory intervention) for primary (blue) and exploratory (gray) end points.

Hand disinfection was performed by 8 of 43 participants (18.6%) in the control group compared with 20 of 52 participants (38.5%) in the intervention group (Table 4; Figure 4). To predict hand disinfection, a logistic regression model using maximum likelihood estimation was fitted (formula: hand disinfection ~ intervention). The explanatory power of the model was weak (Tjur *R*^2^=0.05), which is not uncommon given a single predictor and dichotomous behavioral data. A single environmental cue is not expected to account for the full variance of hygiene behavior, which is shaped by knowledge, attitudes, habits, time pressure, and contextual factors. The relevant question is whether the cue exerts an incremental, practically meaningful effect. The model intercept, corresponding to the control condition (intervention is 0), was −1.48 (*P*<.001). The effect of the intervention was statistically significant (β=1.01, SE 0.49; *P*=.04), corresponding to an OR of 2.73 (95% CI 1.09-7.40). This indicates that the intervention approximately tripled the odds of hand disinfection (Table 5).

**Table 4.** Results of the smell intervention on students’ hygiene behavior from the primary and explorative analyses (N=95).

Implicit objective	NoSmell (n=43), n (%)	Smell (n=52), n (%)	OR (95% CI)	*P* value
Primary outcomes				
Hand disinfection overall	8 (18.6)	20 (38.5)	2.73 (1.09‐7.40)^a^	.04
Use of gloves overall	13 (30.2)	20 (38.5)	1.44 (0.62‐3.45)^a^	.40
Exploratory outcomes				
Initial disinfection	3 (7.0)	2 (3.8)	0.53 (0.07‐3.37)^b^	.66
Initial use of gloves	2 (4.7)	0 (0.0)	0.16 (0.01‐3.38)^b^	.20
Final disinfection	1 (2.3)	10 (19.2)	10.00 (1.80‐187.60)^b^	.01

a Logistic regression.

b For exploratory outcomes, the OR and 95% CI were obtained from logistic regression (with a Haldane-Anscombe correction for an empty cell in initial glove use outcome), and the *P* value was obtained from Fisher exact test.

**Table 5.** Logistic regression models for primary outcomes^a^.

Outcome and parameter	β (SE)	OR (95% CI)	*P* value
Hand disinfection			
Intercept	−1.48 (0.39)	—^b^	<.001
Intervention	1.01 (0.49)	2.73 (1.09-7.40)	.04
Glove use			
Intercept	−0.84 (0.33)	—	.01
Intervention	0.37 (0.44)	1.44 (0.62-3.45)	.40

a Model fit (hand disinfection): Tjur *R*²=0.05; AIC=114.6. Model fit (glove use): Tjur *R*²=0.01; AIC=126.0. AIC refers to Akaike information criterion.

b Not applicable.

Convergent tests supported this finding: Fisher exact test (*P*=.04) and Pearson chi-square test (*χ*^2^_1_=4.47; *P*=.04). The observed effect size was small to medium (Cohen *w*=0.22).

In absolute terms, the intervention increased the probability of hand disinfection by 19.9 percentage points, corresponding to a number needed to treat of 5. In practical terms, this means that for every 5 students exposed to the olfactory intervention, one additional individual would perform hand disinfection who otherwise would not have done so under standard conditions.

#### Use of Gloves

Gloves were used by 13 of 43 participants (30.2%) in the control group, compared with 20 of 52 participants (38.5%) in the intervention group. The logistic regression model predicting glove use (formula: glove use ~ intervention) showed weak explanatory power (Tjur *R*^2^=0.01). The effect of the intervention was not statistically significant (β=0.37, SE 0.44; *P*=.40), corresponding to an OR of 1.44 (95% CI 0.62-3.45). Fisher exact test confirmed the absence of a statistically significant effect (*P*=.52; Table 4; Figure 4). The absolute risk difference was 8.2 percentage points in favor of the intervention group—an effect that could not be distinguished from chance, given the sample size.

#### Sensitivity Analyses

The intervention effect on hand disinfection was robust across all prespecified sensitivity analyses. In the complete-case sample (n=89), the unadjusted OR was 2.81 (95% CI 1.09‐7.75; *P*=.04). Adjustment for sex did not materially change the estimate (adjusted OR=2.58, 95% CI 0.99‐7.20; *P*=.06). The marginally attenuated significance reflects reduced power in the smaller subsample rather than substantive confounding, given the nonsignificant baseline sex imbalance and the stable point estimate. Fisher exact test (*P*=.04) and the chi-square test (*χ*²_1_=4.47; *P*=.04) further supported the finding. For glove use, no sensitivity analysis indicated a statistically significant effect. These convergent results indicate that the primary conclusion is not driven by the choice of analytic sample or by adjustment for baseline covariates.

#### Exploratory End Points

For final hand disinfection, defined as disinfection immediately when leaving the patient environment, a pronounced difference between groups was observed. Only 1 of 43 control participants (2.3%) exhibited this behavior, compared with 10 of 52 participants (19.2%) in the intervention group. Fisher exact test yielded an OR of 9.82 (95% CI 1.29-443.13; *P*=.01). The wide CI reflects the low event rate in the control group and warrants cautious interpretation of this exploratory finding (Figure 3). No statistically significant differences between groups were observed for initial hand disinfection (3/43, 7.0% vs 2/52, 3.8%; OR=0.53, 95% CI 0.07‐3.37; *P*=.66) or for initial glove use (2/43, 4.7% vs 0/52, 0.0%; OR=0.16, 95% CI 0.01‐3.38; *P*=.20).

#### Summary of Findings

Table 4 and Figure 4 present the complete results for all end points, and further information on the analysis is provided in Table 5. The olfactory intervention significantly increased hand disinfection (the first primary end point), whereas glove use (the second primary end point) did not show a statistically significant difference between groups. In the exploratory analyses, the effect on final hand disinfection was particularly pronounced. However, this finding should be interpreted with caution given its exploratory nature and the low event rates observed.

## Discussion

### Impact of Smell on Hand Hygiene and Glove Use

The primary hypothesis, that olfactory cues would increase hand hygiene behavior, was partially supported. Hand disinfection increased significantly under olfactory exposure, whereas glove use showed no significant effect. Furthermore, exposure to the scent of disinfectants increased postcontact hand disinfection, whereas no effects were observed for initial hand disinfection or initial glove use. Because these behavioral changes were observed within a simulated environment, they correspond to Kirkpatrick level 3 (behavior) within simulation and serve as a proxy for, rather than direct evidence of, transfer to real-world clinical practice.

To contextualize these findings, it is important to note that not all hand hygiene behaviors are triggered by the same cues. While initial hand disinfection, which directly targets patient safety, represents a highly trained and routinized behavior, which is done straight as the first action while entering the room, the decision to use gloves is typically reassessed in each situation. This assessment depends on perceived exposure risks, such as contact with bodily fluids—either overt (eg, wound exudate) or subtle (eg, sweat)—as well as the need to protect one’s own skin during wet procedures. These evaluations are guided by sensory impressions.

Our findings suggest that positively valenced cues, such as the clean scent of disinfectants, may reinforce reactive behaviors (eg, final hand disinfection) rather than initiating preventive routines, whereas other behaviors, such as glove use or initial hand hygiene, may require more salient or negatively valenced cues to be affected. Beyond this descriptive pattern, the dissociation between hand disinfection and glove use is itself theoretically informative. The scent of disinfectant carries a strong, repeated real-world association with the act of hand disinfection, whereas no comparable associative link exists for glove use. This selective effect suggests that the olfactory cue operates through specific stimulus-response priming rather than a generalized activation of patient safety behavior, implying that effective sensory augmentation may need to match cues to target behaviors based on learned associations. As glove removal was not possible within the simulation, no conclusions regarding postglove hand hygiene can be drawn from the present data. In line with the existing literature, our findings confirm that positively valenced olfactory cues can also exert a beneficial effect on hand hygiene behavior in VR-based simulations, similar to the effects reported by Birnbach et al and King et al [10,11]. Notably, in contrast to these prior studies, which used a citrus scent, the present study used the smell of a disinfectant as the olfactory stimulus.

In contrast, Schmidtke et al [12] were unable to demonstrate a consistent effect of olfactory priming on hand hygiene behavior in a real-world hospital entrance setting, suggesting that the effectiveness of olfactory cues may depend on contextual specificity and clearly defined patient contact situations. To our knowledge, no prior studies have investigated the influence of olfactory cues on medical glove use, indicating a relevant gap in the current literature.

### Implications for Patient Safety and Simulation Design

Beyond the behavior-specific effects, these results raise broader questions regarding the role of sensory modalities in VR-based patient safety training. Visual and auditory cues are inherently embedded in contemporary VR systems and can be rendered digitally with high fidelity. In contrast, olfactory stimuli cannot be generated virtually and must originate from the physical world. Thus, smell represents the only deliberately introduced real sensory cue in this study, effectively crossing the boundary between virtual and physical experience—a boundary we refer to as the *virtual threshold*.

This concept relates to several established constructs in the simulation and cognitive psychology literature. Presence and embodiment, defined above, both depend on sensory congruence between the simulated scene and learners’ real-world expectations. Ecological validity, in turn, refers to the correspondence between the simulation and the clinical situation it is intended to prepare for. From a memory perspective, the principle of context-dependent retrieval predicts that behaviors learned and habitually performed under specific sensory conditions are more readily activated when those conditions are reinstated. Within this framework, the disinfectant scent can be understood as a sensory retrieval cue that reinstantiates clinically learned associations within an otherwise digitally generated environment, thereby narrowing the gap between virtual and clinical contexts.

Despite its central role in clinical practice, olfaction has largely been neglected in VR-based medical education. As demonstrated in this study, olfactory cues can influence safety-relevant behavior even when visual and auditory cues alone are insufficient. Evidence on olfactory augmentation in immersive medical simulation remains sparse. The most recent systematic review was published in 2016 by Kent et al [23] and primarily investigated olfactory cues in non-VR simulation contexts, leaving the role of real olfactory input in immersive VR-based medical education largely unexplored.

Insights from psychology and entertainment-oriented VR research suggest that olfactory cues can enhance emotional salience, contextual memory, and behavioral realism [24-26]. However, these findings have rarely been translated into medical education, where multisensory perception is fundamental to safe clinical action.

### Theoretical Considerations, Limitations, and Implications for VR-Based Medical Education

This study should be interpreted in light of both theoretical mechanisms and methodological constraints relevant to immersive medical education. Immersive VR aims not only to convey procedural knowledge but also to elicit self-relevant clinical behavior. In this context, it is important to distinguish between immersion (objective system properties), presence (the subjective feeling of “being there”), and embodiment, which refers to the experience of the virtual body as one’s own. Embodiment is commonly conceptualized along the dimensions of body ownership, agency, and self-location and has been identified as a key determinant of behavior in virtual environments, particularly when actions involve self-protection or bodily risk [27-29].

Although olfactory cues do not directly modify visual body representation, they may indirectly reinforce embodiment by strengthening the coupling between bodily actions and real-world physiological expectations. In clinical settings, smells associated with contamination, cleanliness, or disinfection are closely linked to bodily vulnerability and habitual protective routines. Introducing such cues from the physical world into an otherwise fully virtual environment may therefore increase the salience of the virtual body as a potential site of risk. This mechanism could help shift learner behavior from abstract rule compliance toward self-protective, behaviorally realistic responses, such as postcontact hand disinfection. This proposed mechanism was not directly tested in the present study and remains a hypothesis for future investigation.

The relatively low baseline rate of hand disinfection in the control group warrants explicit consideration. Several factors plausibly contribute. First, the novelty of the VR environment may divert attention from routine behaviors. Second, the environmental cues that normally prompt hand hygiene in real settings, such as ambient scent, tactile feedback from the dispenser, and the physical proximity of cleansed surfaces, are absent or only weakly present in a purely audiovisual VR scene. Third, learners may operate with a reduced perceived consequence in an obviously simulated context. Under this reading, the olfactory intervention does not enhance hygiene behavior beyond real-world baselines but rather restores cue-triggered behavior under impoverished sensory conditions. This interpretation does not diminish the practical value of the finding because, if VR environments inherently suppress hygiene-relevant behavior, sensory augmentation is precisely what is required to elicit ecologically valid training responses.

However, several limitations constrain the interpretation and generalizability of these findings. First, the olfactory intervention was limited to a single, positively connoted scent associated with cleanliness. While appropriate for feasibility and safety, this does not reflect the full range of clinically relevant olfactory cues. Future studies should include aversive or negatively valenced odors, which may exert stronger or qualitatively different effects on risk perception and behavior.

Second, participant allocation was constrained by faculty-assigned groups, resulting in limited group sizes and minor imbalances. Although the sample size was sufficient to detect the main effects, these constraints may have reduced statistical precision and may have limited subgroup analyses. Six participants were excluded from the analyses due to missing data, all of whom were in the intervention group, reducing the sample from 95 to 89. Sex distribution showed a nonsignificant imbalance (*P*=.12). Because allocation was quasi-random by room rather than by individual-level randomization, the primary intention-to-treat analysis was complemented by covariate-adjusted sensitivity analyses, and the intervention effect on hand disinfection remained robust across these models. Because participants were allocated within 6 rooms, clustering at the room level cannot be fully excluded, and with only 6 clusters, formal multilevel modeling was not feasible. Third, the behavioral effects observed were behavior-specific: olfactory augmentation influenced overall and postcontact hand disinfection but not initial hand hygiene or glove use. This suggests that olfactory cues may preferentially reinforce reactive, situationally triggered behaviors rather than preventive or purely rule-based routines.

Fourth, the study was conducted within a single VR clinical scenario, which limits extrapolation to other procedures, specialties, or safety behaviors. Broader evaluation across multiple scenarios and end points is required to determine the generalizability of the effects of olfactory augmentation. Finally, technical constraints of the VR tracking system limited the granularity of behavioral measurement. Certain interaction sequences, such as returning to patient contact after hand disinfection outside the tracked examinations, could not be reliably detected, potentially leading to an overestimation of compliance. Future implementations should incorporate more fine-grained temporal and interaction tracking to better capture hygiene-critical behavior.

Fifth, no formal manipulation check was conducted to verify whether participants consciously perceived the olfactory stimulus. We therefore cannot determine whether the effect operates through conscious recognition, such as identifying the disinfectant scent and responding accordingly, or through subconscious priming. Future studies should include a brief postsimulation item assessing scent perception, which would enable stratified analyses of participants who did and did not consciously notice the scent and help adjudicate between conscious and subconscious mechanisms.

A further question concerns the transferability of these findings to experienced clinicians. Our sample comprised medical students with limited clinical experience, whose hygiene behaviors are not yet fully automated. Experienced practitioners might rely on highly routinized motor patterns that are less susceptible to environmental cues, a ceiling interpretation, or they might instead have more strongly encoded cue-behavior associations and therefore respond more readily to congruent sensory primes. Both predictions are plausible and yield different design implications for VR-based continuing education, and which one holds is an empirical question for future studies.

Taken together, these findings and limitations highlight both the promise and the current constraints of olfactory augmentation in VR-based medical education. Future research should systematically vary olfactory valence, improve behavioral tracking fidelity, optimize randomization procedures, and expand scenario diversity. From an educational perspective, the results suggest that olfactory cues may serve as a targeted design element to promote behaviorally realistic, safety-relevant actions in immersive simulations, particularly when the educational goal is not knowledge acquisition alone but the elicitation of authentic clinical behavior.

### Conclusions

This study provides empirical evidence that olfactory cues—an integral yet previously neglected component of clinical environments—can meaningfully enhance behavioral outcomes in immersive VR-based medical education. The introduction of a positively connoted disinfectant scent led to a 19.9 percentage point increase in hand hygiene behavior during simulation training, suggesting that real-world sensory augmentation can strengthen safety-relevant actions beyond purely visual and auditory immersion.

In contrast, glove use was not significantly influenced by the olfactory intervention, indicating that different hygiene behaviors may rely on distinct cognitive mechanisms. While hand disinfection appears susceptible to context-driven sensory priming, glove use may depend more strongly on rule-based or procedural decision processes.

Taken together, these findings highlight the potential of integrating physical sensory cues into digital simulation environments to enhance behavioral realism and patient safety training. Future research should investigate the effects of diverse olfactory stimuli across scenarios and learner populations to further elucidate the mechanisms and generalizability of sensory augmentation in VR-based medical education.

## Supplementary material

10.2196/93148 Checklist 1 CONSORT-EHEALTH (V 1.6.1) checklist.

## Abbreviations

BD/DNC: Brain Death/Death by Neurologic Criteria
CONSORT-EHEALTH: Consolidated Standards of Reporting Trials of Electronic and Mobile Health Applications and Online Telehealth
HMD: head-mounted display
OR: odds ratio
VR: virtual reality
